# Looking with or without seeing in an individual with age-related macular degeneration impairing central vision

**DOI:** 10.1177/20416695241265821

**Published:** 2024-08-14

**Authors:** Li Zhaoping

**Affiliations:** 28328 Max-Planck-Institute for Biological Cybernetics, University of Tübingen, Tübingen, Germany

**Keywords:** attention, object recognition, visual search, reaching/grasping, shapes/objects

## Abstract

Looking leads gaze to objects; seeing recognizes them. Visual crowding makes seeing difficult or impossible before looking brings objects to the fovea. Looking before seeing can be guided by saliency mechanisms in the primary visual cortex (V1). We have proposed that looking and seeing are mainly supported by peripheral and central vision, respectively. This proposal is tested in an observer with central vision loss due to macular degeneration, using a visual search task that can be accomplished solely through looking, but is actually impeded through seeing. The search target is an uniquely oriented, salient, bar among identically shaped bars. Each bar, including the target, is part of an “ "X ” shape. The target’s "X is identical to, although rotated from, the other "X ’s in the image, which normally causes confusion. However, this observer exhibits no such confusion, presumably because she cannot see the "X ’s shape, but can look towards the target. This result demonstrates a critical dichotomy between central and peripheral vision.

Vision involves looking and seeing. Looking shifts gaze and attention to objects; seeing identifies those objects. Typically, looking and seeing are seamlessly coordinated. However, Figure 1 illustrates an example in which they battle. This shows stimulus condition A in a previous study (Zhaoping & Guyader, 2007), along with the characteristic confusion exhibited by normal observers as they searched for an uniquely oriented bar. Crowding in this cluttered image makes seeing difficult before looking. Nevertheless, guided by saliency, gaze reached the target within one second in 50% of the trials in untrained observers (Zhaoping & Guyader, 2007; Zhaoping, 2024). (The target’s unique orientation makes its location salient by V1 mechanisms (Li, 2002).) After seeing the “ "X ” containing the target bar at fovea, gaze abandoned the target in confusion to continue searching elsewhere. This confusion arises from rotational invariance in shape recognition, since this "X is not distinctive from the background "X ’s.

**Figure 1.**
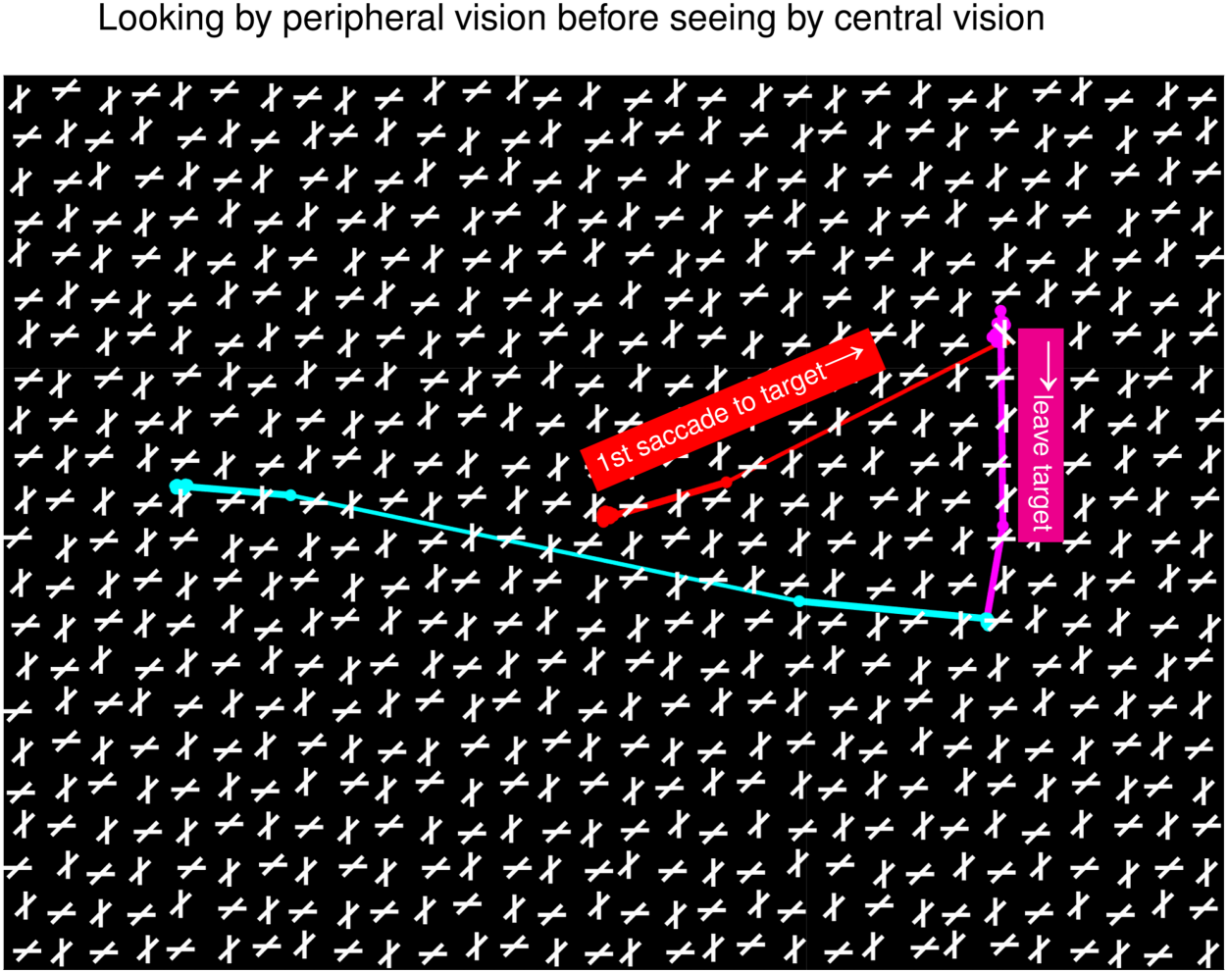
Looking and seeing in a trial of condition A by Zhaoping & Guyader (2007). On top of a search image (spanning $34^{o}\times 46^{o}$ in visual angle), in black and white, is superposed a gaze trajectory (together with explanations) in red, magenta, and cyan, from the start, to the later moments, of a search. The gaze started at the image center when the image appeared. The target, a bar uniquely tilted counterclockwise from vertical, was $15^{o}$ in visual angle from the initial fixation. The first saccade (red) led the gaze to the target—looking. About 0.5 seconds later, the gaze departed from the target (in magenta and cyan). Visual crowding makes the target bar and its associated "X illegible before the looking. Seeing, recognizing this as just another "X , occurred after the gaze reached the target, causing confusion and gaze departure.

Figure 2 shows example search images adapted for the current study for condition A and the control conditions $A_{simple}$ , $B_{simple}$ , and B . In each, the target is an oblique bar tilted either uniquely clockwise or uniquely counterclockwise from vertical. Cardinal, horizontal or vertical, bars only appear in conditions A and B , each intersects an oblique bar to make an "X . In A , all the "X ’s have the same shape, causing the confusion once the target "X is seen. This confusion is absent in B since the target’s "X is distinctively thinner. Zhaoping & Guyader (2007) showed that conditions A and B were equivalent in the time looking needed to gaze at the target for the first time. However, in condition B , the gaze seldom abandoned the target. Hence, a longer response time (RT) to report the target in condition A than B reflects the confusion triggered by seeing the "X ’s shape.

**Figure 2.**
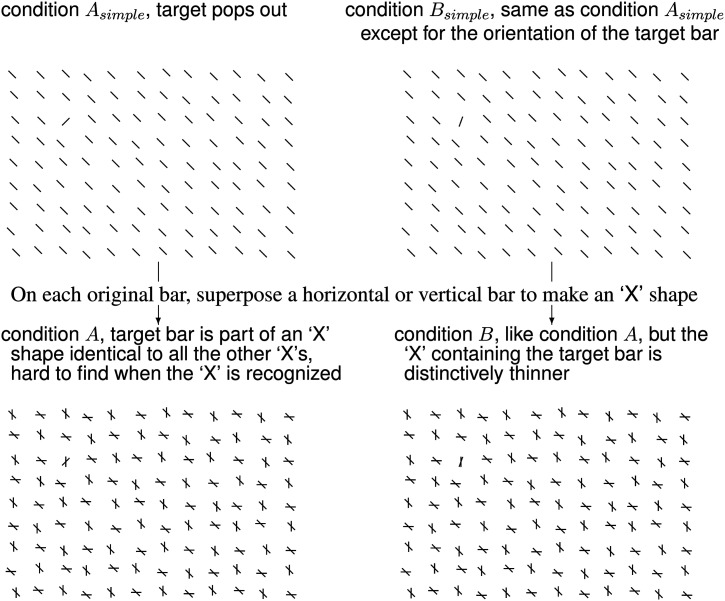
Example search images in four different conditions: $A_{simple}$ , $B_{simple}$ , A , and B . Observers were asked to find a uniquely oriented target bar in each image on a touch-screen display, and touch the target as quickly as possible. In $A_{simple}$ images, all bars are $45^{o}$ clockwise or counterclockwise from vertical; the target is uniquely oriented. Modifying $A_{simple}$ images gives A images, when a horizontal or vertical bar intersects each original bar to make an "X . Modifying A images gives B images, when the target bar’s orientation tilts just $20^{o}$ from the intersecting horizontal/vertical bar. Removing all the horizontal/vertical bars from B images gives $B_{simple}$ images.

Central vision is essential for seeing the target in clutter. Hence, our 86-year-old observer, with her central vision loss due to age-related macular degeneration (MD), may be free from the confusion. She gave up reading ten years ago, but can manage routine housework. She and four age-matched control observers (82 − 88 years old) performed the search on a touch-screen display spanning 21.2×31.5 cm, containing 9 rows × 12 columns of search items. Each observer sat at a self-determined distance (about 50 cm) from the display to perform the task comfortably, making each stimulus bar about $1^{o}$ in length.

The four conditions were randomly interleaved, with 15–20 trials per condition in each experimental session. A search image appeared after observers’ button press, and disappeared once the screen was touched (to report the target) or when 60 seconds had elapsed since its onset. The MD individual complained of not seeing the search items well. Days later, she participated in a second session in which each bar was enlarged by 50% (in length and width) in a sparser (6 rows × 8 columns) search array.

A test trial was deemed successful if the observer touched, within 60 seconds, a screen location no more than 12.5% of the screen’s width/height away from the target’s center horizontally/vertically. RT ( $RT(A_{simple})$ , $RT(B_{simple})$ , RT(A) , and RT(B) ) is the average time needed to touch the screen in the successful trials (Figure 3A). The time-out rate, nonzero only in condition A , is the fraction of the trials without a screen touch within 60 seconds (Figure 3B). Performance accuracy is the fraction ( $F(A_{simple})$ , $F(B_{simple})$ , F(A) , and F(B) ) of the non-time-out trials that were successful (Figure 3C). Each control observer manifested the "X confusion, with RT(A)>RT(B) significantly ( p values p≤.007 from statistical tests) and, except for one control observer, had either substantial time-out rate ( ≥35% ) for condition A or had F(A)<F(B) significantly ( p≤.0008 ).

**Figure 3.**
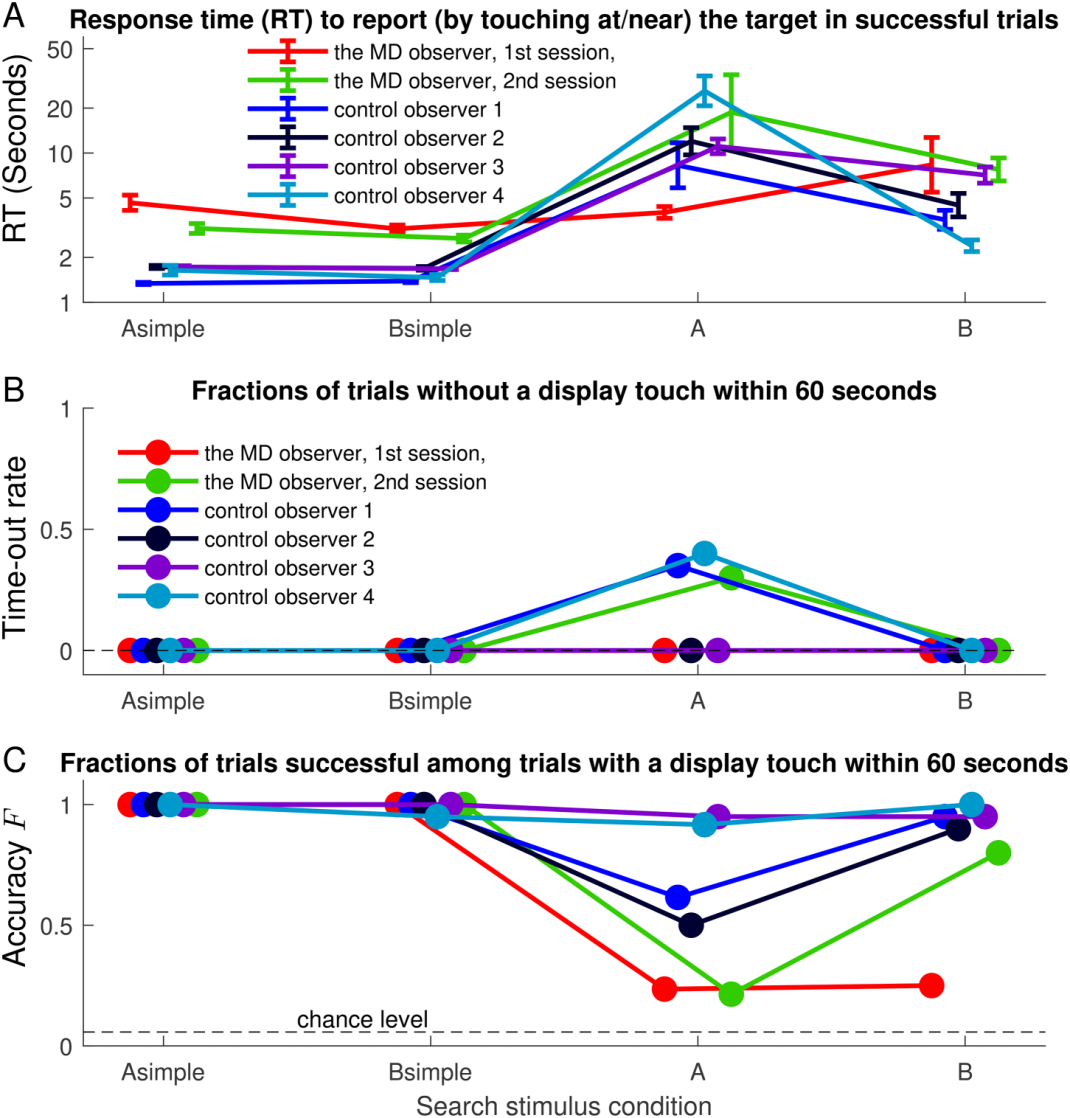
Response time (RTs), time-out rates, and accuracies in the search task.

By contrast, the MD observer exhibited no "X confusion in her first session. She had no time-out trials. Her RT(A)=4.0 seconds was statistically indifferent from her RT(B) ( p=.14 ), $RT(A_{simple})$ ( p=.56 ), and $RT(B_{simple})$ ( p=.07 ). After each RT is normalized (divided) by the observer’s $[RT(A_{simple})+RT(B_{simple})]/2$ , her RT(A)=1.05 was significantly shorter ( p=.002 ) than the average RT(A)=8.3 of the control observers. Her F(A)=24% and F(B)=25% were statistically equivalent ( p=.54 ), and significantly better ( p=.01 ) than 5.8% , the chance level, although worse ( p≤.009 ) than F(A) and F(B) of each control observer. Manifestly, she searched mainly by looking (orienting and touching) without seeing.

In her second session, using enlarged search items in a sparser array, she could see much better, such that her F(B) became statistically equivalent ( p≥.07 ) to the F(B) in three out of four control observers, without changing her RT(B) significantly ( p=.86 ). Consequently, she manifested the "X confusion, with RT(A)>RT(B) ( p=.039 ), F(A)<F(B) ( p=.000 ), a 30% time-out rate for condition A , and a significantly ( p=.007 ) increased (normalized) RT(A)=6.5 (statistically equivalent ( p=.38 ) to the average of the control observers).

In natural behavior, differential functional specializations by central and peripheral vision are obscured, especially with trans-saccadic integration of recognition (Stewart et al., 2020). Our MD individual’s search behavior provides a clearer demonstration that looking and seeing are mainly functions of peripheral and central vision, respectively, as proposed by the Central-peripheral Dichotomy theory (Zhaoping, 2019).
